# The role of primary RPLND in clinical stage IIA/B testicular germ cell tumor: a narrative review

**DOI:** 10.1007/s00120-025-02648-9

**Published:** 2025-08-01

**Authors:** Parth Thakker, Connor Drake, Timothy Masterson, Clint Cary

**Affiliations:** Department of Urology, Indiana University School of Medicine, 340 W 10th St, 46202 Indianapolis, IN USA

**Keywords:** Seminoma/nonseminoma, Primary retroperitoneal lymph node dissection, Germ cell tumor, Relapse, Secondary malignancy, Seminom/Nichtseminom, Primäre retroperitoneale Lymphknotendissektion, Keimzelltumor, Rezidiv, Sekundärmalignom

## Abstract

Retroperitoneal lymph node dissection (RPLND) plays a critical role in the multidisciplinary management of advanced testicular cancer. Cisplatin-based chemotherapy regimens have been the cornerstone of treatment for these patients. Long-term toxicity and secondary malignancy in patients receiving cisplatin, along with surgical advancements, have encouraged providers to revisit the role of primary RPLND for patients with stage IIA/B germ cell tumor. Nerve-sparing primary RPLND offers durable oncologic outcomes without the long-term comorbidities associated with chemotherapy. The focus of this review is to summarize the published data on primary RPLND for stage IIA/B germ cell tumors, to identify the ideal patient population for this treatment modality, and to explore the future of surgical advancements.

## Abstract (de)

Die retroperitoneale Lymphknotendissektion (RPLND) spielt eine entscheidende Rolle bei der multidisziplinären Behandlung von fortgeschrittenem Hodenkrebs. Auf Cisplatin basierende Chemotherapieschemata waren der Eckpfeiler der Behandlung für diese Patienten. Langfristige Toxizität und sekundäre Malignität bei Patienten, die Cisplatin erhalten, sowie chirurgische Fortschritte gaben Veranlassung, die Rolle der primären RPLND bei Patienten mit Keimzelltumoren im Stadium IIA/B zu überdenken. Die nervenschonende primäre RPLND bietet dauerhafte onkologische Ergebnisse ohne die langfristigen Komorbiditäten der Chemotherapie. Der Schwerpunkt dieser Übersichtsarbeit besteht darin, die veröffentlichten Daten zur primären RPLND bei Keimzelltumoren im Stadium IIA/B zusammenzufassen, die ideale Patientenpopulation für diese Behandlungsmodalität zu identifizieren und die Zukunft der chirurgischen Fortschritte zu untersuchen.

## Introduction

Testicular cancer is the most common malignancy in men aged 15–39 years, with the highest incidence seen in individuals of Caucasian and European descent. Although traditionally less common in Asian, Hispanic, and Black African individuals, recent trends have shown an increasing incidence in these groups [1]. Germ cell tumors (GCT) represent 95% of all testicular cancers, which are further divided into seminoma and nonseminoma subsets with almost identical incidence rates [2]. Most patients present with stage I disease and are cured with radical orchiectomy alone; however, roughly 20% of patients have regional retroperitoneal lymphadenopathy [1].

For patients with stage II GCT, systemic platinum-based chemotherapy is a highly effective treatment modality, with disease-free survival rates as high as 90% [3]. The efficacy of chemotherapy, however, comes at a significant cost. In addition to the acute toxicities of platinum-based regimens, the long-term survivors of testicular cancer treated with chemotherapy have an increased risk of developing cardiovascular disease [4]. Hypertension, obesity, and hypercholesterolemia are also found at an increased rate with chemotherapy exposure [5]. Furthermore, the development of secondary malignancy has been reported to be as high as 20% over a cumulative 35-year span [6].

Retroperitoneal lymph node dissection (RPLND) is a treatment modality recommended by the American Urological Association and European Association of Urology for low-volume, marker-negative stage II GCT [7, 8]. For those wishing to avoid the risks of chemotherapy, RPLND offers an alternative treatment modality. Primary PRPLND (pRPLND) has demonstrated success in treating patients, with a 2-year recurrence-free survival (RFS) rate of up to 90% and a postoperative major complication rate as low as 4.5% [9]. Although RPLND has traditionally been reserved for select cases of nonseminomatous germ cell tumor (NSGCT) and post-chemotherapy seminoma, there has been a recent interest in expanding the role of pRPLND for stage II seminoma and NSGCT. Here, we review the role of pRPLND in clinical stage (CS) IIA/B testicular GCT.

## Clinical stage IIA/B NSGCT

Nonseminomatous germ cell tumor encompasses a wide range of histological subtypes, each with unique properties. These subtypes vary in terms of serum tumor marker (STM) pattern, aggressiveness, and chemo- and radio-resistance, making the management of NSGCT highly nuanced. Patients with stage II NSGCT are recommended to undergo 3–4 cycles of upfront chemotherapy. Chemotherapy is preferred by many providers, as historical data indicated that pRPLND resulted in a 70–80% rate of relapse for stage IIA-IIC disease [10, 11]. However, cross-sectional imaging at the time of these reports was in its infancy and modern staging had not been established. As such, disease stage may have been underestimated resulting in highly heterogeneous patient populations and, thus, significant undertreatment. In the modern era, improved imaging and standardized staging has allowed for stage-specific studies, which have shown more durable results for patients undergoing pRPLND, and, as such, pRPLND remains an optional therapeutic modality for CS IIA/B patients with normal STM [12, 13].

Upfront chemotherapy offers a definitive RFS benefit of up to 98% in those treated with cisplatin-based regimens. However, the benefits of pRPLND include accurate pathological staging, avoidance/reduction of chemotherapy, removal of chemo-resistant teratoma, and durable disease-free survival and RFS. Pathological staging obtained during pRPLND provides valuable information to guide further treatment. As an example, Douglawi et al. demonstrated that 35% of patients with CS I disease were upstaged to pathologic stage (PS) II disease during pRPLND. In this study, the 2‑year RFS for patients undergoing pRPLND who had PSII disease without and with adjuvant therapy was 83% and 100%, respectively [14]. This was further corroborated by Tachibana et al. in a study of 117 patients undergoing pRPLND for CS IIA‑C disease. The authors found a 2-year and 5‑year RFS of 80.3% and 79%, respectively, with lymphovascular invasion increasing the risk of recurrence. When stratified by PS, no significant differences in RFS were noted. In this study, 20.6% of patients experienced a relapse, were treated with adjuvant chemotherapy, and remained disease free [15]. Furthermore, by selecting ideal candidates for pRPLND in CS IIA/B disease, the outcomes with pRPLND may be further improved. A study performed at Memorial Sloan Kettering demonstrated that by selecting patients with unifocal disease, lymph nodes ≤2 cm, no metastasis outside the primary landing zone, and normal STMs, the 5‑year RFS improved from 78% to 100% with a sustained disease-specific survival ranging from 96% to 100%. In this study, however, 35% of patients had pN2‑3, extranodal extension, or they desired adjuvant treatment and were treated with 2 cycles of etoposide and platinum chemotherapy [16, 17].

Teratoma is a unique subset of NSGCT that is generally chemo- and radioresistant and most often presents with normal STM. Malignant transformation and growing teratoma can lead to catastrophic scenarios in patients with unresected teratoma. Foster et al. demonstrated that teratoma can be found in the retroperitoneum in 85.6% of patients when any teratoma is present in the testis [18]. This concept was further elaborated by Cary et al., who found that 100% of patients with pure teratoma of the testis had teratoma in the retroperitoneum identified at pRPLND [19]. As such, providers may be wary of patients with cystic-appearing retroperitoneal masses, normal STM, and those with teratoma within the testis, preferentially treating those patients with pRPLND.

## Clinical stage IIA/B seminoma

Testicular seminoma has a unique history due to its radio- and chemosensitivity. The comparative surgical morbidity in the 1980s and oncologic efficacy of noninvasive treatment modalities led to the sidelining of RPLND in the management of patients with stage II seminoma. As the understanding of long-term morbidity and cytotoxic effects of radio- and chemotherapy has developed, a renewed interest in pRPLND for these patients has arisen. The feasibility of pRPLND for CS IIA/B seminoma was demonstrated by several retrospective studies from high-volume centers. In a study of 67 patients undergoing unilateral or bilateral template pRPLND, Tachibana et al. found a 2-year RFS of 80.2%, which improved to 93.7% in those that had CS I and progressed to CS II disease after the first year of surveillance [20]. Similar findings were reported by Matulewicz et al., who found a 2-year RFS of 81% with no in-field relapses [21].

Phase III studies, randomizing patients to pRPLND vs. chemotherapy or radiation, would be near-impossible to perform. Several phase II studies, however, have built upon the interest of pRPLND in CS II seminoma (Table 1). The PRIMETEST study enrolled 33 patients of whom a subset received 1 cycle of carboplatin with subsequent relapse. The 2‑year RFS was 70% in this patient population, although it notably included patients with maximal tumor size of 5 cm. Furthermore, all patients in PRIMETEST were treated with a unilateral template pRPLND, and in 40% of patients who experienced a relapse, the contralateral retroperitoneum was involved [22]. The COTRIMS trial had a similar schema, where both patients with CS IIA/B and those with progressive CSI disease were included. All patients in COTRIMS received a unilateral template RPLND with only one patient presenting with a node >4 cm. Heidenreich et al. found a 3-year RFS of 88% with no in-field recurrence and one contralateral retroperitoneal lymph node recurrence [23]. Similarly, in the multi-institutional SEMS trial of 55 patients, where both unilateral and bilateral template dissections were allowed, an 81% 2‑year RFS was noted. Seven (58%) cases of relapse were in the retroperitoneum with 25% in the contralateral retroperitoneum for those receiving unilateral template dissections. Notably, the SEMS trial limited inclusion to patients with a preoperative retroperitoneal mass size of < 3 cm. However, 35% of patients had pathologic disease >3 cm, indicating a significant proportion of patients had more bulky disease than anticipated [24].

**Table 1:** Inclusion criteria for enrollment, clinical and pathological features, surgical details, and outcomes for pRPLND in stage IIA/B seminoma, stratified by investigation

	COTRIMS	SEMS	PRIMETEST	SWENOTECA	Indiana
*N*	34	55	33	62	67
Inclusion LN size (cm)	<5	<3	<5	≤3	<5
Clinical stage, *n* (%)					
*I AS*	11 (37)	36 (65)	19 (58)	33 (53)	48 (72)
*I* *+* *carbo*	0 (0)	0 (0)	5 (15)	6 (10)	0 (0)
*IIA/B*	19 (63)	19 (35)	9 (27)	23 (37)	19 (28)
Pathological stage, *n* (%)					
*IIA*	19 (63)	40 (73)	13 (39)	60 (97)	26 (39)
*IIB*	11 (37)	15 (27)	20 (61)	0 (0)	41 (61)
pN0 rate, *n* (%)	3 (10)	9 (16)	3 (9)	2 (3.2)	1 (1.5)
2‑year RFS, %	88	81	70	90	80
Follow-up (mo)	21	33	32	23	22
Surgical approach, *n* (%)					
*oRPLND*	27 (90)	55 (100)	14 (42)	22 (35)	67 (100)
*rRPLND*	3 (10)	0 (0)	19 (58)	40 (65)	0 (0)
Template, *n* (%)					
*Bilateral*	0 (0)	19 (35)	0 (0)	2 (3)	31 (46)
*Unilateral*	34 (100)	36 (65)	33 (100)	60 (97)	36 (54)
LOS (days)	4.5	3	6	2	3
Operative time (min)	125	233	169	139	205
EBL (cc)	<150	150	50	100	150
Major complication, *n* (%)	4 (13.3)	4 (7)	3 (9)	5 (8)	3 (4.5)

*Major complication* Clavien–Dindo ≥3, *CS* clinical stage, *AS* active surveillance, *LN* lymph node, *RFS* relapse-free survival, *mo* months, *RPLND* retroperitoneal lymph node dissection (*o* open, *r* robotic), *LOS* length of stay, *EBL* estimated blood loss

Of all the studies in this space, the SWENOTECA study had the most heterogeneous patient population including patients with CSIIA/B, CSI with relapse on surveillance, and CSI with relapse after 1 cycle of carboplatin. Like the SEMS trial, patients with multifocal retroperitoneal masses and those with nodes >3 cm were excluded. In this study of 62 patients, a 2-year RFS of 90% was noted. Six patients experienced a relapse and four (67%) of these patients had an in-field relapse, while the remaining two (33%) were out-of-field; however it is unclear whether those may have been prevented by performing bilateral template pRPLND [25]. Furthermore, 18 patients (29%) received adjuvant chemotherapy following pRPLND in this study. To elucidate the role of modified template pRPLND in CS IIA/B seminoma, Zeng et al. performed a mapping study investigating the rate of crossover in these patients. The investigators found a microscopic disease crossover rate of 6–11.1% in the contralateral landing zone with a 2-year RFS of 80.6% vs. 86.6% for unilateral vs. bilateral templates, respectively [26]. Although not statistically different, the use of bilateral templates in this setting was supported by data from Memorial Sloan Kettering [21]. The SEMS trial, however, did not demonstrate a difference in recurrence patterns between unilateral and bilateral template dissections. In view of the somewhat conflicting results, further studies are required to understand the true efficacy of unilateral template dissection in this setting.

## Surgical approach

Open RPLND (oRPLND) is the traditional approach to RPLND in testicular GCT. As with most procedures, surgeons seek to minimize surgical morbidity while maintaining oncologic efficacy. Starting with prostatectomy, followed by partial and radical nephrectomy, and then cystectomy, the robotic approach has been progressively utilized for these purposes, with much success. Conversely, there is a paucity of data regarding robotic RPLND (rRPLND) for testicular GCT. Various modifications of patient positioning have been described. Initially the flank positioning was described, as this was the most familiar approach to retroperitoneal surgery; however, only unilateral RPLND was possible. Modification of the approach to a supine position allowed for unilateral and bilateral RPLND without repositioning [27]. Several series have been published regarding the safety, feasibility, and early oncologic efficacy of rRPLND; however, most of these studies have been small case-series [28]. One of the larger series compared the RFS and perioperative outcomes between surgical approaches. In this propensity-score matching study of 178 patients, relapse rates were similar between the groups, with the oRPLND group having larger metastatic deposits (3 vs. 1.7 cm) and half of the required operative time (4.3 vs. 8.8 h) compared to rRPLND. The latter approach offered benefits in terms of blood loss (300 vs. 200 cc) and length of stay (5 vs. 1 day; [29]). In-field relapse rates in these groups were not significantly different. Additionally, the total cost of surgery and postoperative stay for the two approaches were shown to be equivalent in a population-based study [30]. The feasibility of rRPLND has been corroborated by Pongratanakul et al. in a matched-pair analysis of the surgical approaches. The authors found similar RFS and operative times between oRPLND and rRPLND but a significantly longer length of stay in the open group (6 vs. 3 days; [31]). While the benefits of rRPLND lie primarily in length of stay, the length of stay in open cohorts was longer than can be expected, and thus this may represent an overestimation of the true length of stay in oRPLND. While these studies offer promise in uncomplicated cases, the utility of nationwide database and propensity-score matching studies is limited, as these techniques lack granular data and are unable to remove unknown selection biases. Furthermore, reports of earlier discharge using an open, extraperitoneal approach and blood loss of 100–250 cc in phase II studies continue to support the open approach as standard of care [21–24]. While rRPLND appears to have potential in the management of stage IIA/B patients, prospective trials and larger studies are required prior to offering rRPLND as a standard-of-care option in the management of these patients. Comparative, prospective trials evaluating the open and robotic approaches are required to understand the true oncologic outcomes and perioperative benefits.

## Long-term toxicity of chemotherapy

Chemotherapy remains the most common modality for most patients with metastatic CS IIA/B GCT. The goal of pRPLND for these patients is to provide an efficient route to cure while minimizing morbidity and need for adjuvant therapy. Patients with testicular GCT have long life expectancies and, as such, chemotherapy-associated morbidity is particularly relevant. The risk of developing cardiovascular morbidity was clearly demonstrated in a study by Meinardi et al. They found that compared to CSI patients on surveillance, chemotherapy patients had higher rates of hypertension, hypercholesterolemia, triglyceridemia, and insulin resistance in addition to high rates of microalbuminuria, Raynaud’s phenomenon, and left ventricular dysfunction [32].

In addition to cardiovascular effects, secondary malignancies (SM) remain a concern after chemotherapy in long-term survivors, particularly those receiving cisplatin-based regimens. As cisplatin is detected in various organs for years after completion, SM may be anticipated. Chemotherapy receipt has been associated with increased risks of developing solid-organ malignancies including mesothelial, colon, bladder, stomach, and hematologic malignancies. The risk of developing SM may remain elevated for 35 years after chemotherapy [6]. A combination of bleomycin-induced pulmonary toxicity, cisplatin-induced renal toxicity, and cardiotoxicity may increase the mortality rate in patients receiving chemotherapy. Among risk factors for SM and toxicities is the cumulative dose of cisplatin; therefore, reducing chemotherapy may be prudent. While receipt of adjuvant chemotherapy may be considered treatment failure, patients receiving chemotherapy in the adjuvant setting typically receive fewer cumulative chemotherapeutic agents and thus may experience less toxicity. Further investigations regarding differences in long-term toxicity among these patient populations may be warranted.

## Practical conclusion

- Primary retroperitoneal lymph node dissection (RPLND) in stage IIA/B germ cell tumors (GCT) has gained traction in recent years as a way to provide durable cure rates while minimizing morbidity and mortality.
- Offering a 2-year recurrence-free survival (RFS) of 70–90% for seminoma and up to 83% for nonseminomatous GCT with an acceptable complication rate, chemotherapy may be avoided in a significant proportion of patients.
- Ideal patients for primary RPLND have low-volume, unifocal disease.
- The efficacy of modified template dissections in pure seminoma remains mixed, given the recent results of phase II trials and mapping studies.

## Declarations

For this article no studies with human participants or animals were performed by any of the authors. All studies mentioned were in accordance with the ethical standards indicated in each case.
